# Atrial Fibrillation as a Marker of Occult Cancer

**DOI:** 10.1371/journal.pone.0102861

**Published:** 2014-08-13

**Authors:** Eva B. Ostenfeld, Rune Erichsen, Lars Pedersen, Dóra K. Farkas, Noel S. Weiss, Henrik T. Sørensen

**Affiliations:** 1 Department of Clinical Epidemiology, Aarhus University Hospital, Aarhus N, Denmark; 2 Department of Gastrointestinal Surgery, Aalborg University Hospital, Aalborg, Denmark; 3 Department of Epidemiology, University of Washington, Seattle, Washington, United States of America; Indiana University School of Medicine, United States of America

## Abstract

**Background:**

Recent studies suggest that cancer increases risk of atrial fibrillation. Whether atrial fibrillation is a marker for underlying occult cancer is unknown.

**Methods:**

We conducted a cohort study (1980–2011) of all Danish patients with new-onset atrial fibrillation. To examine cancer risk, we computed absolute risk at 3 months and standardized incidence ratios (SIRs) by comparing observed cancer incidence among patients newly diagnosed with atrial fibrillation with that expected based on national cancer incidence during the period.

**Results:**

Median follow-up time was 3.4 years among 269 742 atrial fibrillation patients. Within 3 months of follow-up, 6656 cancers occurred (absolute risk, 2.5%; 95% confidence intervals [CI], 2.4%–2.5%) versus 1302 expected, yielding a SIR of 5.11; 95% CI, 4.99–5.24. Associations were particularly strong for cancers of the lung, kidney, colon, ovary, and for non-Hodgkin's lymphoma. The SIR within 3 months of follow-up was 7.02; 95% CI, 6.76–7.28 for metastatic and 3.53; 95% CI, 3.38–3.68 for localized cancer. Beyond 3 months of follow-up, overall cancer risk was modestly increased (SIR, 1.13; 95% CI, 1.12–1.15).

**Conclusion:**

Patients with new-onset atrial fibrillation had a markedly increased relative risk of a cancer diagnosis within the next three months, however, corresponding absolute risk was small.

## Introduction

Atrial fibrillation is a common cardiac arrhythmia: the lifetime risk of at least one episode is more than 20% [1]. Well-established risk factors for atrial fibrillation include older age, male gender, and presence of coronary and valvular heart disease, heart failure, hypertension, diabetes, obesity, hyperthyroidism, alcohol abuse, and smoking [2], [3].

Cancer may also increase risk of atrial fibrillation, but few data exist for this association. Two single-center case-control studies have reported a higher prevalence of atrial fibrillation among colorectal and breast cancer patients compared to controls [4], [5]. Recently, another case-control study confirmed the association between colorectal cancer and atrial fibrillation or flutter [6]. Suggested pathogenic mechanisms underlying these findings included inflammation and autonomic, endocrine, and coagulation alterations associated with cancer [4]–[6].

Given an association between cancer and atrial fibrillation, we posed the question whether atrial fibrillation could be an early sign of the presence of occult cancer. Cancer is the leading cause of death in industrialized countries [7] and early detection may improve treatment possibilities and prognosis [8]. We therefore examined risk of localized or metastatic cancer following an atrial fibrillation diagnosis in a population-based cohort using data from Danish medical registries.

## Materials And Methods

We conducted this cohort study in the setting of the entire Danish population of 7 920 831 people between January 1, 1980 and December 31, 2011 [9]. Since 1968, all Danish residents have been assigned a unique personal identifier by the Civil Registration System. This 10-digit number encodes age and sex, and allows unambiguous individual-level data linkage between registries. The Civil Registration System records residence, vital status (dead/alive), and date of death and is updated daily.

### Patients with atrial fibrillation

We used the Danish National Registry of Patients to identify all persons with a first inpatient or outpatient diagnosis of atrial fibrillation during the study period. This registry was established in 1977 and contains records of 99% of all non-psychiatric hospitalizations in Denmark. Since 1995, the Registry of Patients has also included all outpatient clinic and emergency room visits. Registry information includes dates of admission and discharge, surgical and diagnostic procedures, and up to 20 discharge diagnoses, coded by physicians according to the 8^th^ revision of the *International Classification of Diseases* (ICD-8) until the end of 1993 and the 10^th^ revision (ICD-10) thereafter [10]. We also included patients with atrial flutter since this condition is coded together with atrial fibrillation in the ICD-10 (see Codes S1) [11]. However, approximately 94% of patients with the combined diagnosis have atrial fibrillation (11). To minimize risk of including recurrent atrial fibrillation cases during the first years after the establishment of the Registry of Patients, the study period began in 1980.

We also retrieved data from the Registry of Patients on conditions associated with atrial fibrillation. We included covariates such as cardiovascular disease, diabetes, hyperthyroidism, obesity, chronic obstructive pulmonary disease (as proxy for smoking), and alcoholism recorded prior to the atrial fibrillation diagnosis (index date), as well as surgical procedures performed within three months prior to the index date (see Codes S1). We used the Charlson Comorbidity Index to measure the burden of comorbidity, excluding cancer since it was our outcome of interest. This index is based on disease categories, each weighted according to its impact on one-year mortality [12], [13]. After excluding cancer diagnoses from the index (see Table S1 for codes), we defined three levels of comorbidity: low (Charlson score  =  0), medium (Charlson score  =  1–2), and high (Charlson score  =  3+).

### Cancer risk

To identify incident cancer cases, the identities of all atrial fibrillation patients were linked to the Danish Cancer Registry, which has recorded cases of incident cancer since 1943 (now reclassified according to the ICD-10 coding system) [14]. We grouped the cancers according to Appendix 9 of the National Board of Health's annual cancer report (The Danish Health and Medicines Authority, 2011) [15] (see Codes S1) and also in categories related to smoking, alcohol, or obesity [16] (see Table S2). From the Cancer Registry, we obtained additional information on stage at diagnosis and classified cancers as “localized” or “metastatic”. Patients with a cancer diagnosis, including non-melanoma skin cancer, prior to the date of their atrial fibrillation diagnosis were excluded.

### Statistical analyses

We followed each patient for the occurrence of cancer from the date of their atrial fibrillation diagnosis until death, emigration, or December 31, 2011, whichever came first. We computed absolute risk of cancer at 3 months (cumulative incidence) treating death as a competing risk [17]. We used SIRs as a measure of relative risk, comparing observed cancer incidence among patients with atrial fibrillation with that expected based on cancer incidence in the Danish population. Expected numbers of cancer cases were calculated based on national incidence rates by age (one-year groups), sex, and calendar period (one-year intervals). Multiplying the number of person-years of observation by the national incidence rates yielded the number of cancer patients that would be expected if patients with atrial fibrillation had the same risk of cancer as the general population. Confidence intervals (CIs) for the SIRs were computed under the assumption that the observed number of cases in a specific category followed a Poisson distribution. Exact 95% CIs were used when the observed number was less than ten, otherwise, Byar's approximation was used [17]. To examine variations in cancer risk following atrial fibrillation in the presence of associated conditions, SIRs were calculated in strata of gender, age at diagnosis (in age groups of 0–29 years, 30–49 years, 50–69 years, and 70+ years), the atrial fibrillation risk factors noted above, and Charlson comorbidity index scores.

We then divided follow-up time into two periods (one to three months following an atrial fibrillation diagnosis and more than three months following this diagnosis), considering cancers diagnosed during the first period as prevalent occult cancers. We computed overall and site-specific SIRs within each time period and separate SIRs for localized and metastatic cancer.

To avoid including pre-existing atrial fibrillation cases identified during the diagnostic work-up for cancer, we conducted two subsequent analyses. First, we restricted the analysis to patients with atrial fibrillation listed as their primary diagnosis (i.e. the main cause of the hospitalization). Second, we excluded patients who received diagnoses of both atrial fibrillation and cancer during the same hospitalization or outpatient visit.

Analyses were performed using SAS, version 9.2 (SAS Institute Inc., Cary, North Carolina, USA). The study was approved by the Central Region of Denmark (record number 1-16-02-1-08). According to the Danish Data Protection Law, registry-based studies require no ethics board approval.

## Results

We identified 269 742 patients with a new diagnosis of atrial fibrillation during the 1980–2011 period (Table 1). There were more males (53%) than females (47%) and median age at time of atrial fibrillation diagnosis was 74 years. Median follow-up time was 3.4 years (interquartile range, 1.0–7.3 years). Within 3 months of follow-up, 6656 patients with atrial fibrillation were diagnosed with cancer yielding an absolute risk of 2.5%; 95% CI, 2.4%–2.5%).

**Table 1:** Standardized incidence ratios for all cancers diagnosed following atrial fibrillation, stratified according to covariates, Denmark, 1980–2011.^a^

Patients with atrial fibrillation	No. (%)	Observed/expected number of cancers	SIR (95% CI)
All	269 742 (100)	37 869/28 864	1.31 (1.30–1.33)
Women	126 808 (47)	15 134/11 479	1.32 (1.30–1.34)
Men	142 934 (53)	22 735/17 385	1.31 (1.29–1.32)
Age at diagnosis (yrs)			
0–29	2168 (1)	27/28	0.95 (0.63–1.38)
30–49	14 716 (5)	1040/833	1.25 (1.17–1.33)
50–69	84 367 (31)	14 127/10 882	1.30 (1.28–1.33)
70+	168 491 (62)	22 675/17 120	1.32 (1.31–1.34)
Cardiovascular disease	98 147 (36)	11 730/9135	1.28 (1.26–1.31)
Diabetes	20 265 (8)	2104/1544	1.36 (1.31–1.42)
Hyperthyroidism	6016 (2)	751/556	1.35 (1.26–1.45)
Chronic obstructive pulmonary disease	23 295 (9)	2742/1654	1.66 (1.60–1.72)
Obesity	9875 (35)	1100/793	1.39 (1.31–1.47)
Alcoholism	6861 (3)	759/456	1.66 (1.55–1.79)
Charlson score 0	156 283 (58)	24 324/19 024	1.28 (1.26–1.29)
Charlson score 1–2	92 249 (34)	11 644/8547	1.36 (1.34–1.39)
Charlson score 3+	21 210 (8)	1901/1292	1.47 (1.41–1.54)
Surgical procedure within past 3 months	29 677 (11)	4647/2888	1.61 (1.56–1.66)

a SIR, standardized incidence ratio; CI, confidence interval.

The SIR for all types of cancers throughout the follow-up period was 1.31; 95% CI, 1.30–1.33), based on 37 869 observed and 28 864 expected cancer cases. We found no noteworthy differences in SIRs by gender or across the age groups from 30–49 years to 70+ years, except that patients aged 0 to 29 years had a lower relative risk of cancer (SIR, 0.95; 95% CI, 0.63–1.38). Patients with a history of alcoholism (SIR, 1.66; 95% CI, 1.55–1.79), chronic obstructive pulmonary disease (SIR, 1.66; 95% CI, 1.60–1.72), surgical procedures within 3 months prior to an atrial fibrillation diagnosis (SIR, 1.61; 95% CI, 1.56–1.66) or a Charlson score of 3+ (SIR, 1.47; 95% CI, 1.41–1.54) had the highest SIRs (Table 1).

**Figure 1:**
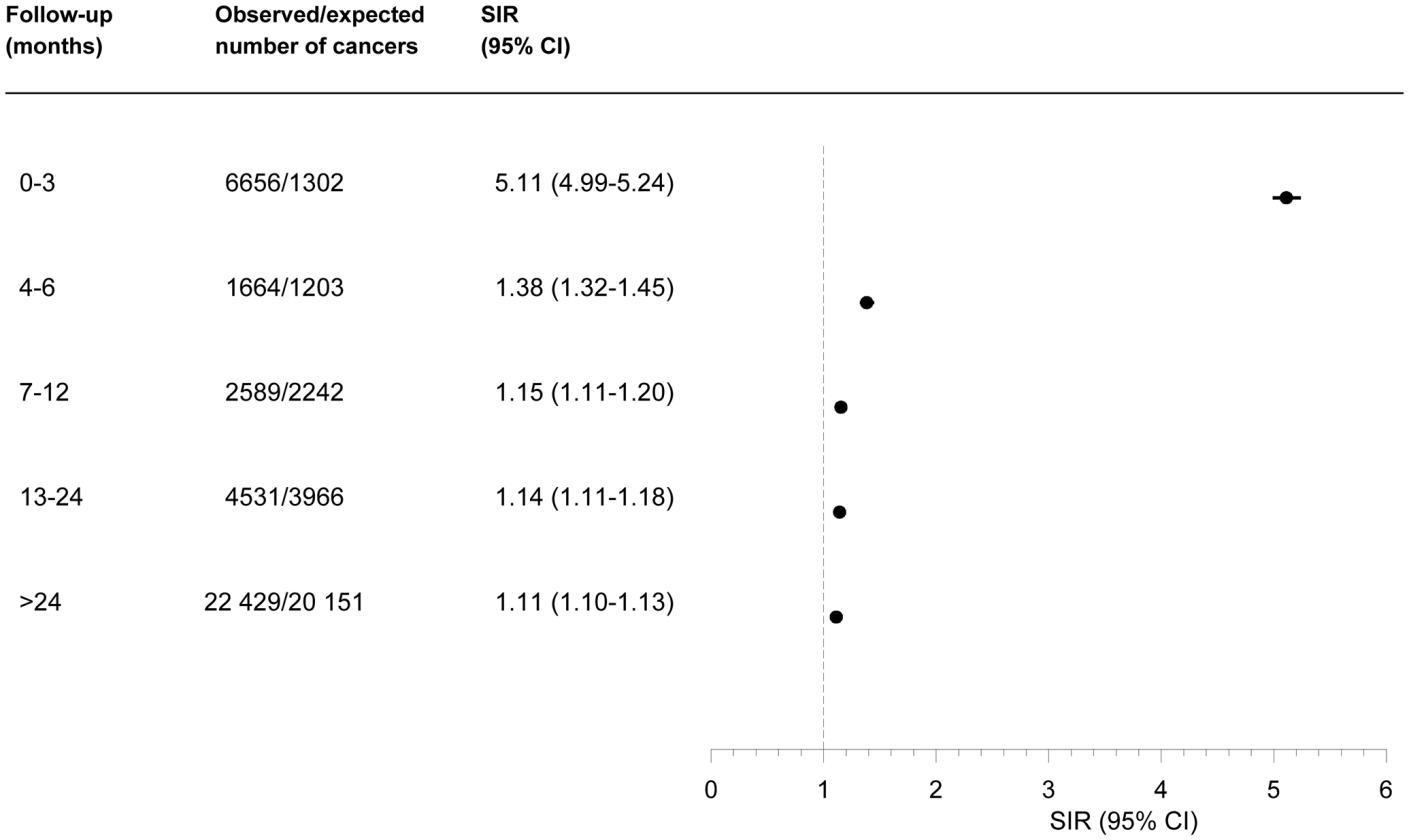
Standardized incidence ratios (SIRs) and 95% confidence intervals (CIs) for all cancer sites following atrial fibrillation by follow-up period, Denmark, 1980–2011.

The relative risk of a cancer diagnosis was clearly elevated shortly after atrial fibrillation diagnosis (Figure 1). Table 2 shows the SIRs for overall and site-specific cancers according to follow-up time. The SIR within 3 months of follow-up was 5.11; 95% CI, 4.99–5.24 based on the 6656 diagnosed cancers versus 1302 expected cancers. SIRs for all types of cancer were increased in this period, but most pronounced for cancers of the lung, kidney and colon. Also, risk of non-Hodgkin's lymphoma was markedly increased, as was risk of cancers related to smoking (SIR, 7.29; 95% CI, 7.07–7.52) and obesity (SIR, 7.05; 95% CI: 6.67–7.44). In contrast, basal cell carcinoma risk was only slightly increased. Beyond 3 months of follow-up, overall and site-specific cancer occurrence was only modestly (though persistently) increased (Table 2).

**Table 2:** Standardized incidence ratios for major cancer sites or lifestyle-related cancers diagnosed following atrial fibrillation by follow-up period, Denmark, 1980–2011.^a^^,^ b

	0 to 3 months		More than 3 months	
Site or type of cancer	Observed/expected number of cancers	SIR (95% CI)	Observed/expected number of cancers	SIR (95% CI)
All cancers	6656/1302	5.11 (4.99–5.24)	31 213/27 562	1.13 (1.12–1.15)
Esophagus	83/15	5.53 (4.41–6.86)	394/325	1.21 (1.10–1.34)
Stomach	154/29	5.37 (4.56–6.29)	589/537	1.10 (1.01–1.19)
Colon	876/115	7.59 (7.10–8.11)	2699/2386	1.13 (1.09–1.17)
Rectum	301/56	5.39 (4.80–6.03)	1160/1152	1.01 (0.95–1.07)
Pancreas	173/35	4.90 (4.20–5.69)	838/722	1.16 (1.08–1.24)
Lung, bronchi and trachea	1719/147	11.72 (11.18–12.29)	3542/3060	1.16 (1.12–1.20)
Breast	374/96	3.89 (3.50–4.30)	2239/1933	1.16 (1.11–1.21)
Uterus	112/20	5.49 (4.52–6.61)	502/398	1.26 (1.15–1.38)
Ovary	102/16	6.50 (5.30–7.89)	317/300	1.06 (0.94–1.18)
Uterine cervix	28/7	3.82 (2.54–5.52)	123/133	0.92 (0.77–1.10)
Prostate	444/135	3.29 (2.99–3.61)	3186/3075	1.04 (1.01–1.07)
Kidney	160/20	7.88 (6.71–9.20)	623/419	1.49 (1.37–1.61)
Urinary bladder	291/78	3.74 (3.32–4.20)	1822/1620	1.12 (1.07–1.18)
Brain	111/19	5.88 (4.83–7.08)	448/406	1.10 (1.00–1.21)
Non-Hodgkin lymphoma	270/41	6.58 (5.82–7.41)	1049/870	1.21 (1.13–1.28)
Malignant melanoma	54/24	2.21 (1.66–2.88)	624/548	1.14 (1.05–1.23)
Basal cell carcinoma	243/211	1.15 (1.01–1.31)	5370/4712	1.14 (1.11–1.17)
Tobacco-related cancers	4157/570	7.29 (7.07–7.52)	13 305/11 731	1.13 (1.11–1.15)
Alcohol-related cancers	1761/315	5.59 (5.33–5.85)	7299/6484	1.13 (1.10–1.15)
Obesity-related cancers	1294/184	7.05 (6.67–7.44)	4227/3770	1.20 (1.17–1.24)

a SIR, standardized incidence ratio; CI, confidence interval.

b Because only the 13 most incident cancers for each gender (The Danish Health and Medicines Authority, 2010) are shown, the number of cases for the individual sites and types do not add up to the total number.

Data for cancer spread at the time of diagnosis were available for 26 528 (78%) of the 34,962 atrial fibrillation cases. Within the first 3 months of follow-up, 2848 metastatic cancers were diagnosed as compared to 406 expected (SIR, 7.02; 95% CI, 6.76–7.28). The corresponding SIR for localized cancer was 3.53; 95% CI, 3.38–3.68, based on 2129 observed and 603 expected cases.

In the analysis restricted to the 150 552 patients with atrial fibrillation recorded as their primary diagnosis, 2365 cancers were observed within the first 3 months of follow-up. Only 757 cases were expected, yielding a SIR of 3.13; 95% CI, 3.00–3.25. The corresponding absolute risk was 1.5%; 95% CI, 1.4–1.5. After excluding patients who received diagnoses of atrial fibrillation and cancer during the same hospitalization or outpatient visit, the 3-month SIR was 1.63; 95% CI, 1.56–1.70 and the absolute risk was 0.8%; 95% CI, 0.77%–0.84%. After 3 months, relative cancer risk estimates were virtually identical to corresponding SIRs provided in Table 2.

## Discussion

In this nationwide cohort study of patients with new-onset atrial fibrillation, we observed a markedly increased relative risk of a cancer diagnosis within the first 3 months following a diagnosis of atrial fibrillation. We observed particularly strong associations between atrial fibrillation and cancers of the lung, kidney and colon. Moreover, atrial fibrillation was strongly associated with metastatic cancer. Still, 3-month absolute cancer risk was only 2.5%. Beyond 3 months, the overall relative cancer risk was only slightly increased.

The rapid fall in relative risks after the initial 3 months of follow-up suggests that the cancers were likely to be present at the time of the atrial fibrillation diagnosis rather than arising as a consequence of atrial fibrillation. It is plausible that heightened medical surveillance among patients with newly diagnosed atrial fibrillation influenced our findings. Diagnostic work-up for atrial fibrillation or conditions caused by atrial fibrillation includes thorough clinical examinations and screening for underlying diseases and may lead to cancer detection. For instance, a chest x-ray may reveal preclinical lung cancer, and cerebral scan may reveal preclinical brain cancer in patients with suspected stroke as a complication of atrial fibrillation. However, incident diagnoses of basal cell carcinoma, which would be expected to be very sensitive to detection bias, were quite stable during initial follow-up after the atrial fibrillation event. Also, if diagnostic bias were a major factor contributing to increased cancer risk at 3 months, we would expect a compensatory decline in cancer risk in the subsequent follow-up period, which was not observed. The association between atrial fibrillation and subsequent cancer was particularly strong for advanced cancers, providing further evidence that our results were not driven solely by detection bias. However, shared risk factors may explain the observed associations in part. Hypertension and diabetes predispose to atrial fibrillation [2] and cancer [18], [19], as do smoking, alcoholism, and obesity [2], [16], [20]–[22]. The increased risk of lifestyle-related cancers, in particular smoking-related cancers (*e.g*., lung and kidney cancers) found in the present study, supports this supposition.

Our findings indicated that occult cancer was likely to be present at the time of the atrial fibrillation diagnosis, which is in accordance with the few available studies examining associations between existing cancer and atrial fibrillation risk [4]–[6], [23]. In the Italian single-center case-control study, the prevalence of atrial fibrillation was two times higher among patients admitted for colorectal or breast cancer surgery compared to patients undergoing non-cancer-related surgery (Guzzetti *et al*, 2008). Moreover, in the case control study including 28,333 atrial fibrillation cases and 283,260 sex, age, and county-matched controls, the odds ratio associating atrial fibrillation and colorectal cancer was 11.8; 95% CI: 9.3 to 14.9 within 90 days of the diagnosis, and very similar results were found for other cancers (Erichsen *et al*, 2011).

Cancer may cause atrial fibrillation through associated systemic factors. Inflammation, determined by elevations in C-reactive protein and related biomarkers, has been associated with the presence of atrial fibrillation and future development of atrial fibrillation [24], [25]. Although a causal relation remains unclear, inflammation may induce structural and electrical remodeling of the atria leading to atrial fibrillation [24]. Also, hypercoagulability attributable to cancer [26], [27] may trigger atrial fibrillation through pulmonary micro-embolism (3).

The strengths of our study include its population-based design within the setting of a uniform tax-supported health care system. Our study population was well defined with complete follow up [28], [29] Although coding errors in the registries may occur, the positive predictive value of atrial fibrillation has been reported to be 93% (11). The accuracy of major diagnoses in the Cancer Registry is similarly high [28]. Nonetheless, misclassification might occur. Patients diagnosed with atrial fibrillation during a diagnostic work-up for cancer could have been included in our cohort erroneously, leading to overestimation of relative risks. Still, the associations persisted when the cohort was restricted to patients whose atrial fibrillation was recorded as a primary diagnosis during a hospital contact prior to the cancer diagnosis. When we excluded patients diagnosed with both atrial fibrillation and cancer during the same hospitalization, relative risks may even have been underestimated because more atrial fibrillation patients would have been evaluated for presence of cancer (with negative findings), compared to the general population. By study design, we excluded patients with a cancer diagnosis prior to their atrial fibrillation diagnosis. We were not able to apply a similar restriction to the general population, which may have been contaminated with prevalent cancer cases. Finally, we had no data on atrial fibrillation diagnosed and treated solely by general practitioners. However, most patients with atrial fibrillation are examined by cardiologists in the hospital or in a hospital outpatient clinic, and thus have associated records in the Registry of Patients [30].

Our findings may have clinical implications. In our cohort, most of the cancers that were found during the first 3 months of follow-up were likely present and undetected at the time of atrial fibrillation diagnosis. Of these, 57% had metastases. Still, searching for malignancies at atrial fibrillation diagnosis might have required extensive work-up, and it is unclear whether earlier diagnosis would have changed the prognosis.

In conclusion, we found, that patients with new-onset atrial fibrillation had a markedly increased probability of a cancer diagnosis within 3 months after atrial fibrillation diagnosis. Moreover, atrial fibrillation was strongly associated with metastatic cancer. Beyond 3 months, however, the relative cancer risk was only modestly elevated.

## Supporting Information

Codes S1 (DOCX)

Table S1 **ICD codes defining a modified Charlson Comorbidity Index.**
(DOCX)

Table S2 **Categories of cancers related to tobacco, alcohol, and obesity.**
(DOCX)
