# Supplementary material for: Atrial Fibrillation as a Marker of Occult Cancer
Source: PLoS One. 2014 Aug 13;9(8):e102861. doi: 10.1371/journal.pone.0102861 (PMC4138009; doi:10.1371/journal.pone.0102861)
Supplement: Codes S1 — (DOCX) [file pone.0102861.s001.docx]

**Codes S1**

***International Classification of Diseases* (ICD) codes defining atrial fibrillation or flutter**

ICD-8: 427.93-427.94 and ICD-10: I48

**ICD codes defining cancer**

ICD-10: <http://www.sst.dk/publ/Publ2011/DAF/Cancer/Cancerregisteret2010.pdf>

(Appendix 9, any C-code, page 42, on)

**ICD codes defining comorbidity or surgical procedures**

**Cardiovascular diseases including hypertension**

a. Heart valvular diseases: ICD-8: 393-397, 424; ICD-10: I05-08, I34-37, I39, I51.1A

b. Hypertension: ICD-8: 400-404; ICD-10: I10-15

c. Coronary artery diseases: ICD-8: 410-414; ICD-10: I20-25

d. Cardiac failure: ICD-8: 427.09-427.11, 427.19; ICD-10: I50

**Diabetes:** ICD-8: 249, 250; ICD-10: E10 - E14

**Hyperthyroidism:** ICD-8: 242; ICD-10: E05

**Chronic obstructive pulmonary disease:** ICD-8: 491, 492; ICD-10: J41-J44

**Obesity:** ICD-8: 277.99; ICD-10: E65-E66

**Alcoholism:** ICD-8: 291, 303 (except 303.90), 571.09, 571.10, 577.10, 979, 980**;** ICD-10: F10 (except F10.0), G31.2, G62.1, G72.1, I42.6, K29.2, K86.0, K70, R78.0, T51, Z72.1

**Surgical procedures:** Before 1996: opr; From 1996: K
