# Supplementary material for: Atrial Fibrillation as a Marker of Occult Cancer
Source: PLoS One. 2014 Aug 13;9(8):e102861. doi: 10.1371/journal.pone.0102861 (PMC4138009; doi:10.1371/journal.pone.0102861)
Supplement: Table S1 — ICD codes defining a modified Charlson Comorbidity Index. (DOCX) [file pone.0102861.s002.docx]

| **Table S1.** ICD codes defining a modified Charlson Comorbidity Index | | | |
| --- | --- | --- | --- |
| **Disease** | **ICD-8** | **ICD-10** | **Score** |
| Myocardial infarction | 410 | I21;I22;I23 | 1 |
| Congestive heart failure | 427.09; 427.10; 427.11; 427.19; 428.99; 782.49 | I50; I11.0; I13.0; I13.2 | 1 |
| Peripheral vascular disease | 440; 441; 442; 443; 444; 445 | I70; I71; I72; I73; I74; I77 | 1 |
| Cerebrovascular disease | 430-438 | I60-I69; G45; G46 | 1 |
| Dementia | 290.09-290.19; 293.09 | F00-F03; F05.1; G30 | 1 |
| Chronic pulmonary disease | 490-493; 515-518 | J40-J47; J60-J67; J68.4; J70.1 | 1 |
| Connective tissue disease | 712; 716; 734; 446; 135.99 | J70.3; J84.1; J92.0; J96.1; J98.2; J98.3 | 1 |
| Ulcer disease | 530.91; 530.98; 531-534 | M05; M06; M08; M09;M30;M31; | 1 |
| Mild liver disease | 571; 573.01; 573.04 | M32; M33; M34; M35; M36; D86 | 1 |
| Diabetes type 1/type 2 | 249.00; 249.06; 249.07; 249.09 | K22.1; K25-K28 | 1 |
| Hemiplegia | 250.00; 250.06; 250.07; 250.09 | B18; K70.0-K70.3; K70.9; K71; K73; K74; K76.0 | 2 |
| Moderate to severe renal disease | 344 | E10.0, E10.1; E10.9 | 2 |
| Diabetes with end organ damage type 1/type 2 | 403; 404; 580-583; 584; 590.09; 593.19; 753.10-753.19; 792 | E11.0; E11.1; E11.9 | 2 |
| Moderate/severe liver disease |  | G81; G82 | 3 |
| AIDS | 249.01-249.05; 249.08 | I12; I13; N00-N05; N07; N11; N14; N17-N19; Q61 | 6 |
