# Supplementary material for: Atrial Fibrillation as a Marker of Occult Cancer
Source: PLoS One. 2014 Aug 13;9(8):e102861. doi: 10.1371/journal.pone.0102861 (PMC4138009; doi:10.1371/journal.pone.0102861)
Supplement: Table S2 — Categories of cancers related to tobacco, alcohol, and obesity. (DOCX) [file pone.0102861.s003.docx]

| **Table S2.** Categories of cancers related to tobacco, alcohol, and obesity | | |
| --- | --- | --- |
| **Tobacco** | **Alcohol** | **Obesity** |
| Oral cavity | Oral cavity | Colon |
| Pharynx | Pharynx | Thyroid |
| Larynx | Larynx | Gall Bladder |
| Esophagus | Esophagus | Endometrial |
| Stomach | Liver | Kidney |
| Colon | Colon | Esophagus |
| Rectum | Rectum |  |
| Liver | Breast |  |
| Pancreas |  |  |
| Nasal Cavity and Paranasal Sinuses |  |  |
| Larynx |  |  |
| Lung, bronchi and trachea |  |  |
| Uterine cervix |  |  |
| Ovary |  |  |
| Bladder |  |  |
| Kidney |  |  |
